# Low-grade appendiceal mucinous neoplasms confined to the appendix: clinical manifestations and CT findings

**DOI:** 10.1136/jim-2018-000975

**Published:** 2019-07-11

**Authors:** Xiang-Rong Yu, Jun Mao, Wei Tang, Xiang-ying Meng, Ye Tian, Zhong-Li Du

**Affiliations:** 1 Department of Radiology, Zhuhai Hospital of Jinan University, Zhuhai People’s Hospital, Zhuhai, China; 2 Department of Radiology, Fudan University Shanghai Cancer Center, Shanghai, China; 3 Department of Pharmaceutics, Key Laboratory of Smart Drug Delivery, Ministry of Education, School of Pharmacy, Fudan University, Shanghai, China

**Keywords:** low-grade mucinous neoplasm, appendix, computed tomography, diagnosis

## Abstract

The clinical findings and CT images are investigated in order to fulfill an early preoperative diagnosis and increase awareness of low-grade appendiceal mucinous neoplasm (LAMN) confined to the appendix. 17 cases with histologically proven LAMNs confined to the appendix were included in this study. All patients had received multiphase CT examinations before the surgery. The imaging criteria included shape, size, margin, attenuation, secondary degeneration and internal mass enhancement pattern. In CT images, all cases appeared as oval or tubular cystic masses (average attenuation 20.4±3.6 Hounsfield units), with the longest dimensions ranging from approximately 38 to 106 mm (mean 66.3 mm), and the ratio of length against width was 1.83 in average. The cystic wall was unevenly thickened, with a mean maximal wall thickness of 5.7 mm (>10 mm in 3 cases). The inner capsule wall was rough, and calcification was observed in 3 cases. A few amounts of periappendiceal fat stranding were noted in 2 cases. Mild ring mural enhancement of the cystic wall was seen during the arterial phase, with progressive enhancement during the portal venous phase. In addition, mini enhancing mural nodules was observed in 5 cases. Although preoperative diagnosis of LAMNs confined to the appendix remains challenging, it should be considered when a focal well-defined cystic mass with slightly higher than water attenuation, thickened cystic wall with ring mural enhancement and a characteristic progressive contrast enhancement in CT imaging, especially in older females with non-specific symptoms similar to appendicitis.

**Significance of this study**

**What is already known about this subject?**

- Low-grade appendiceal mucinous neoplasms (LAMNs) confined to the appendiceal lumen do not show definitive malignant features, they can proliferate outside the appendix in a malignant fashion and result in the development of pseudomyxoma peritonei, a life-threatening complication with 45% 10-year survival.
- Considering uncertain potential malignant progression, an early and accurate preoperative identification of LAMNs confined to the appendix is crucial for decisions of prognosis and treatment strategy.
- Prospective clinical diagnosis is often difficult because the presentations of LAMNs are quite variable, vague and non-specific.

**What are the new findings?**

- In this work, we evaluated the CT characteristics and described the clinical features of LAMNs confined to the appendix to assess the feasibility of a preoperative diagnosis for therapy.
- In CT images, LAMNs confined to the appendix presented as unilocular cystic masses in the right lower quadrant of the abdomen.
- The largest diameter of masses ranged from approximately 38 to 106 mm (mean 66.3mm) in our series, and the ratio of length against width was 1.83 in average.
- The cystic wall was thickened with a mean maximal wall thickness of 5.7 mm (3–14 mm in range).
- On multiphase CT images, early mild ring mural enhancement of the cystic wall was seen during the arterial phase, with a 48.8 Hounsfield units (HU) CT attenuation in average (38–65 HU in range).
- Progressive contrast enhancement occurred in the portal venous phases with 66.0 HU CT attenuation in average (45–84 HU in range).

**Significance of this study**

**How might these results change the focus of research or clinical practice?**

- Preoperative detection of LAMNs confined to the appendix is important because of treatment implications.
- Sensitive detection and adequate diagnosis of LAMNs confined to the appendix will improve the efficacy of the treatment, and allow for better prediction of the prognosis and outcome after therapy.
- Although preoperative diagnosis remains challenging, we believe that a combination of clinical manifestations and suggestive findings on CT imaging makes it possible to improve the diagnostic accuracy of appendiceal neoplasms.

## Introduction

Appendiceal mucinous neoplasms are rare entities by <1% of appendectomies in a whole.^1–3^ In the past, the frequent disparities between the histological findings and clinical behaviour accounted for controversial histological classification of mucinous neoplasms.^4^ In 2003, first classification was made by Misdraji *et al* into low-grade appendiceal mucinous neoplasms (LAMNs) and mucinous adenocarcinoma based on complexity of architecture and degree of cytological atypia,^5^ which was adopted by the World Health Organization classification in 2010.^6^ Even though LAMNs confined to the appendiceal lumen do not show definitive malignant features, they can proliferate outside the appendix in a malignant fashion and result in the development of pseudomyxoma peritonei, a life-threatening complication with 45% 10-year survival.^4^ Considerable uncertain potential malignant progression, an early and accurate preoperative identification of LAMNs confined to the appendix is crucial for decisions of prognosis and treatment strategy. To date, although numerous studies have been conducted on the histopathological and histogenetic characteristics of LAMNs,^1–4^ only sporadic cases have been reported on the imaging findings.^7 8^ In this work, we evaluated the CT characteristics and described the clinical features of LAMNs confined to the appendix to assess the feasibility of a preoperative diagnosis for therapy.

## Materials and methods

Seventeen cases with surgically and histologically proven LAMNs confined to the appendix were chosen in this study by reviewing documented abdominal CT examinations in the radiological database from December 2011 to October 2016.

All patients received non-contrast CT and contrast-enhanced scan under a GE Light Speed 64-slice spiral CT machine. CT scan was performed in breath holding state with following imaging parameters: 1.25 mm section thickness reconstructions, 120 kV voltage, automatic dose modulation of 90–270 mA tube current and 256×256 matrix. An intravenous bolus dose of 100 mL of a non-ionic iodinated contrast agent (Optiray 320, TycoHealthcare, Quebec, Canada) was administrated through the antecubital vein at a rate of 3–4 mL/s by high pressure syringe. Regions of interest set on the reference image of the descending aorta were scanned under an automatic trigger scanning mode with a 5 s delay and 200 Hounsfield units (HU) of the preset CT number. Multiphase contrast-enhanced CT were obtained under this mode 18–30 s (arterial phase), and 60–80 s (parenchymal phase) after contrast agent injection; 1.5 hours before CT scan, all patients orally received 1500 mL of 2.0% diatrizoate meglumine solution during an approximately 45 min interval for standardization and uniform distention of small bowel.^9^

The CT images were read and analyzed double-blindly by two senior radiologists with over 10-year experience in imaging diagnosis. The images were specifically evaluated for lesion location, size, shape, margin, attenuation, pattern and degree of enhancement, cyst formation, maximal wall thickness, internal septations, calcification, adjacent fat stranding, enlarged lymph nodes, etc. Additional clinical data, such as age, sex, symptoms, duration and preoperative biochemical workup including white blood cells (WBC) counts and tumor markers studies, were also reviewed.

All patients received surgical right hemicolectomy and were treated with additional cytoreductive surgery with hyperthermic intraperitoneal chemotherapy based on pathological results. Time intervals between the preoperative CT examination and surgery varied from 2 to 5 days. Histopathological examination of all lesions was performed on specimens obtained during surgery.

## Results

The preoperative clinical and imaging findings of all patients are summarized in tables 1–3. The median age of the patients was 58.6 years (37–88 years in range) with a male against female ratio of 1:1.4. The most common presentation was right lower quadrant pain in 14 cases, with durations ranging from 5 days to 2 years (median, 3 months), 5 cases of which were initially suspected of acute appendicitis. Among the other patients presented here included four cases with a tender and mobile mass on palpation. The rest three cases were asymptomatic, and were discovered incidentally during a routine health ultrasound examination. Laboratory results showed five cases with mild leukocytosis with increasing neutrophil count, while two cases with slightly raised levels of related tumor markers carcinoembryonic antigen (CEA), and carbohydrate antigen (CA) 19-9 level.

**Table 1:** Clinical findings in 17 patients with LAMNs confined to the appendix

Case/sex/age	Clinical presentation	Symptom duration	WBC counts	Tumor markers level
1/F/71	Abdominal pain with a mass in the right iliac fossa	6 months	Normal	Normal
2/F/88	Acute appendicitis	5 days	Mild leukocytosis	Normal
3/F/48	Right lower quadrant pain	1 year	Normal	Normal
4/F/50	Acute appendicitis	2 weeks	Mild leukocytosis	Normal
5/F/67	Incidental finding	–	Normal	Normal
6/M/65	Abdominal pain with a mass in the right iliac fossa	10 months	Normal	Normal
7/M/49	Incidental finding	–	Normal	Normal
8/F/54	Right lower quadrant pain	2 years	Normal	Normal
9/M/65	Acute appendicitis	2 weeks	Mild leukocytosis	Normal
10/F/59	Abdominal pain with a mass in the right iliac fossa	3 months	Normal	Normal
11/F/49	Acute appendicitis	10 days	Mild leukocytosis	Normal
12/F/54	Incidental finding	–	Normal	Normal
13/M/65	Abdominal pain and change in bowel habits	3 months	Normal	Slightly elevated
14/M/61	Abdominal pain, anorexia and weight loss	1 year	Normal	Slightly elevated
15/F/58	Acute appendicitis	1 week	Mild leukocytosis	Normal
16/M/37	Abdominal pain with a mass in the right iliac fossa	4 months	Normal	Normal
17/M/56	Abdominal pain, nausea and vomiting	2 weeks	Normal	Normal

age, year; F, female; LAMN, low-grade appendiceal mucinous neoplasm; M, male.

**Table 2:** CT findings in 17 patients with LAMNs confined to the appendix

Case/sex/age	Maximum diameter	Length/width	Morphology	Margin	Cystic attenuation	Maximal cystic wall	Calcification
1/F/71	65	1.96	Oval	Well-circumscribed	15 HU	3	-
2/F/88	66	1.69	Oval	Well-circumscribed	20 HU	3	+
3/F/48	68	1.70	Oval	Well-circumscribed	22 HU	7	-
4/F/50	76	1.91	Oval	Well-circumscribed	21 HU	5	-
5/F/67	66	1.69	Oval	Well-circumscribed	18 HU	3	-
6/M/65	39	1.52	Oval	Well-circumscribed	25 HU	10	-
7/M/49	106	2.30	Tubular	Well-circumscribed	16 HU	13	+
8/F/54	52	1.87	Oval	Well-circumscribed	21 HU	5	+
9/M/65	58	1.78	Oval	Well-circumscribed	22 HU	3	-
10/F/59	81	2.25	Oval	Well-circumscribed	18 HU	4	-
11/F/49	38	1.58	Oval	Well-circumscribed	21 HU	5	-
12/F/54	100	2.32	Tubular	Well-circumscribed	20 HU	5	-
13/M/65	65	1.91	Oval	Well-circumscribed	17 HU	14	-
14/M/61	66	1.73	Oval	Well-circumscribed	29 HU	7	-
15/F/58	66	1.65	Oval	Well-circumscribed	18 HU	3	-
16/M/37	70	1.67	Oval	Well-circumscribed	19 HU	3	-
17/M/56	45	1.56	Oval	Well-circumscribed	25 HU	4	-

age, year; F, female; LAMN, low-grade appendiceal mucinous neoplasm; M, male; mass size unit, mm.

**Table 3:** The CT values of cystic wall on an unenhanced, arterial phase and parenchymal phase images of LAMNs

Case/sex/age	Unenhanced phase	Arterial phase	Parenchymal phase	Mural nodules
1/F/71	38 HU	55 HU	81 HU	+
2/F/88	43 HU	58 HU	84 HU	+
3/F/48	32 HU	48 HU	62 HU	-
4/F/50	25 HU	39 HU	51 HU	-
5/F/67	28 HU	45 HU	60 HU	-
6/M/65	38 HU	60 HU	77 HU	+
7/M/49	43 HU	65 HU	82 HU	+
8/F/54	21 HU	39 HU	51 HU	-
9/M/65	32 HU	48 HU	62 HU	-
10/F/59	38 HU	46 HU	71 HU	+
11/F/49	31 HU	47 HU	69 HU	-
12/F/54	40 HU	51 HU	70 HU	-
13/M/65	27 HU	41 HU	58 HU	-
14/M/61	37 HU	51 HU	69 HU	-
15/F/58	38 HU	48 HU	61 HU	-
16/M/37	39 HU	51 HU	69 HU	-
17/M/56	20 HU	38 HU	45 HU	-

age, year; F, female; LAMN, low-grade appendiceal mucinous neoplasm; M, male.

**Figure 1:**
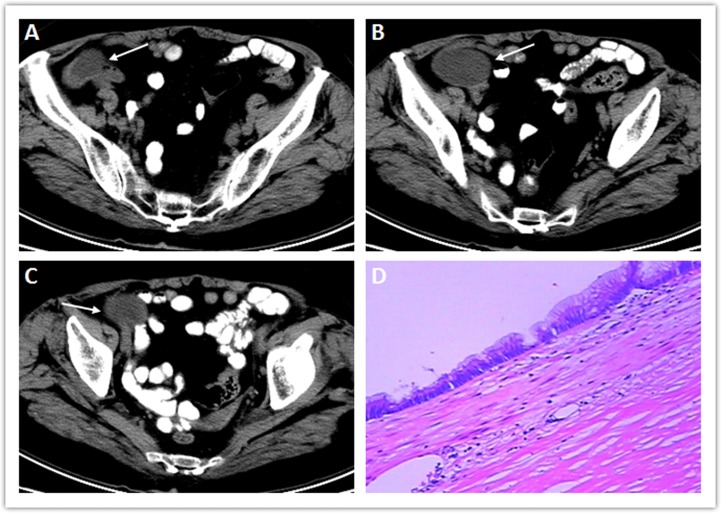
A well-circumscribed tubular cystic mass (arrow) in the right iliac fossa (A–C), displacing the adjacent bowel. The mean CT value of the intratumoral cystic contents was 27 HU. No significant solid component inside the mass was shown on CT. Mucosa reduced to single layer of flat mucinous epithelium with apical mucin droplets and pseudostratified hyperchromatic nuclei (D).

**Figure 2:**
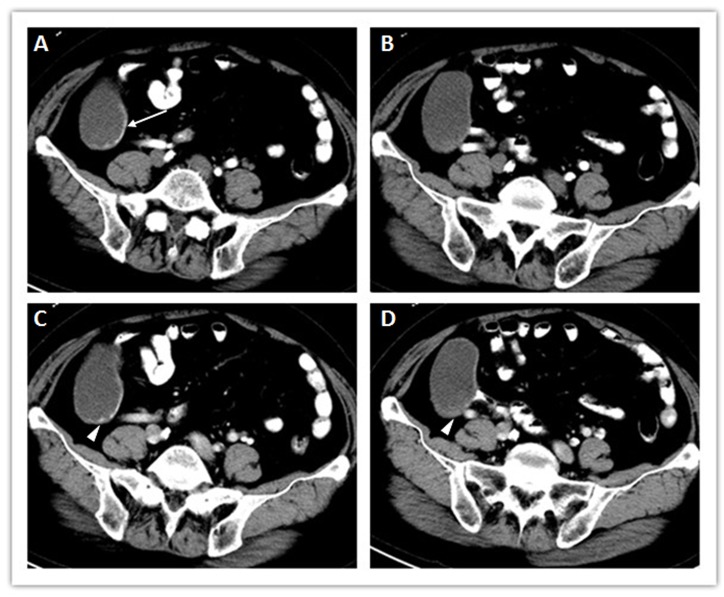
A well-defined appendiceal lesion with curvilinear mural calcifications (arrow) and intact margins (A). The cystic wall was unevenly thickened with slight enhancement in the arterial phase (B), and progressive enhancement in the portal venous phase (C-D). Meanwhile, the mini enhancing mural nodule (C), and the thickened wall (D) were evident in the portal venous phase.

**Figure 3:**
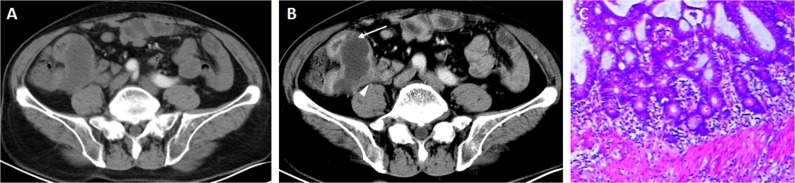
Contrast enhanced CT scan showed cystic mass with mild ring mural enhancement in the arterial phase (A). Progressive enhancement of mini mural nodule (arrow), and a few amounts of periappendiceal fat stranding (arrow head) were noted in the portal venous phase (B). The mucinous epithelium was remarkably bland with small nuclei with no signs of invasive growth (C).

In our study, LAMNs confined to the appendix presented as unilocular cystic masses in the right lower quadrant of the abdomen. The largest diameter of masses ranged from approximately 38 to 106 mm (mean=64 mm) in our series, and the ratio of length against width was 1.83 in average. The mass wasoval in 15 cases (88.2%) and tubular in 2 cases (11.8%). All tumors were encapsulated, showing well-circumscribed margins (figures 1–3). The unenhanced CT values of the intratumoral cystic contents ranged from 15 to 29 HU (mean 20.4±3.6 HU). Besides, the cystic wall was thickened with a mean maximal wall thickness of 5.7 mm (3–14 mm in range). Although cyst wall showed inhomogeneous thickness, the outer capsule wall was yet regular and smooth while the inner capsule wall was rough. Calcification was observed in three cases related to the cyst wall (figure 3). On multiphase CT images, early mild ring mural enhancement of the cystic wall was seen during the arterial phase, with a 48.8 HU CT attenuation in average (38–65 HU in range). Progressive contrast enhancement occurred in the portal venous phases with 66.0 HU CT attenuation in average (45–84 HU in range) (figure 2). Mini and enhancing mural nodules were observed in five cases with no significant solid components inside the tumor shown on the CT. A few amounts of periappendiceal fat stranding were noted in two cases without abscess. Adjacent bowel was compressed and displaced by the mass in eight cases without any invasion of the tumor to adjacent structures shown on the CT. All patients in our study were demonstrated as localized diseases without involvement of regional lymph node and presence of mucinous peritoneal carcinomatosis.

In this study, all patients received laparotomic right hemicolectomy, during which the surface of the cystic masses from the appendix was found to be tense and smooth. The masses were resected fairly easily off the surrounding tissue. The surgical manipulations were very gentle and the cystic masses remained intact during the procedure without leakage into the peritoneal cavity. The resected specimen was in oval shape with 3–6 mm thickening fibrous capsule with translucent mucoid ﬂuid in lumen. Non-invasive glands with mucin were seen on the outer appendiceal wall and peritoneal cavity in all cases. Microscopic manifestation of all specimens was characterized by a proliferation of high columnar glandular epithelium with mild atypia and mitotic that secreted mucus. Meanwhile, the underlying lymphoid stroma atrophied or even disappeared. Fibrotic and thinning tissue replaced the muscularis propia with dystrophic calcification in three cases and neutrophil infiltration in five cases. Moreover, no regional lymph nodes were observed and no mucinous peritoneal carcinomatosis occurred. These findings were consistent with the diagnosis of LAMNs without extraluminal infiltration. The postoperative course was smooth, and no further surgical therapy or adjuvant chemotherapy was required in all patients. Follow-up with CT scans every 6 months and surveillance of CEA and CA 19-9 tumor markers have been conducted, showing a favorable outcome currently without evidence of recurrence.

## Discussion

Histologically, a villous or flat proliferation of mucinous epithelium with low-grade atypia was a hallmark of the LAMNs with common mucin production in examination.^5 10^ Even though the LAMNs without definitive malignant features pursue a predictable clinical course in general, the precursor lesions to pseudomyxoma peritonei cannot be ruled out.^1^ Because prominent mucin production increases intraluminal pressure, which may penetrate into or through the appendix wall, subsequently disseminate to the peritoneal cavity, and cause mucinous ascites ultimately.

To the best of our knowledge, total excision of LAMNs confined to the appendix is associated with a favorable outcome.^5 11^ We report here 17 cases of LAMNs that were successfully treated with straightforward right hemicolectomy without disease recurrence postoperatively. Previous research demonstrated that LAMNs confined to the appendix provide better long-term survival rates than extra-appendiceal spread cases.^11^ No recurrence was seen in patients with LAMNs confined to the appendix, whereas 3-year, 5-year and 10-year survival rates in LAMNs with extra-appendiceal spread were 100%, 86% and 45%, respectively.^5^ Moreover, the traditional treatment of LAMNs confined to the appendix is right hemicolectomy. If there is spillage of mucin, additional cytoreductive surgery and hyperthermic intraperitoneal chemotherapy should be taken into account to improve outcome and long-term survival for appendiceal pseudomyxoma peritonei.^12–14^

Hence, given the potential malignant progression, sensitive detection and adequate diagnosis of LAMNs confined to the appendix will improve the efficacy of the treatment, and allow for better prediction of the prognosis and outcome after therapy. However, prospective clinical diagnosis is often difficult because the presentations of LAMNs are quite variable, vague and non-specific. In the present study, three patients (17.6%) were clinically silent and discovered incidentally without symptom, and most cases initially presented as right lower quadrant pain simulating appendicitis, possibly associated with the presence of a palpable mass in the right iliac fossa. Other symptoms included anorexia, nausea, vomiting, weight loss and change in bowel habits, which were related to the compression mass exerted on adjacent structures. The median duration of symptoms was 3 months in the present study, which suggested that the indolent nature of LAMNs often led to chronic appendiceal obstruction and a late diagnosis. Meanwhile, due to these vague symptoms of abdominal distension and pain, many LAMNs confined to the appendix were often overlooked and misdiagnosed as appendicitis. The clinical features in this study showed high similarity to those in previous reports,^3 8 12^ providing further evidence that symptomatology is unable to diagnosis preoperatively alone.

Although occurring in all age groups, LAMNs are more commonly seen in the fifth and sixth decades of life, with a high occurrence rate in women.^1 15–18^ In a retrospective study of 98 patients by Fournier *et al*, 57% of patients were females.^1^ As observed in our study, the median age of the 17 patients was 58.6 (37–88 years in range) with a male against female ratio of 1:1.4, in accordance with previous reports.^1^ Therefore, in female population over 50 with clinical symptom of abdominal pain in the right iliac fossa, diagnosis of acute appendicitis should be accompanied by awareness of LAMNs as differential diagnosis.

In the preoperative laboratory investigation, WBC counts were not significantly higher in patients with LAMNs at admission. Mild leukocytosis with increase of neutrophil count occurred in five cases, suggesting very slight inflammation of the appendiceal wall. Furthermore, patients with LAMNs confined to appendix often showed lower levels of CEA, CA 19-9 and CA 125 preoperatively. As observed in our study, slightly increased serum levels of related tumor markers occurred only in two cases. However, it is highly recommended to include these markers in diagnostic investigations because previous reports found an intriguing observation that LAMNs with significant increase of tumor marker levels were at higher risk of disease recurrence or death, indicating that closer monitoring or intervention appears to be warranted.^1 14 19^

CT has been proved a fast and ubiquitous modality used to evaluate intra-abdominal organs for preoperative workup and an important role during the staging process in routine clinical practice. The healthy appendix on CT imaging appears as thin-walled tubular or annular structure wrapped by the mesenteric fat in the right lower abdomen with the diameter generally <6 mm. Imaging findings associated with appendicitis have been widely documented in the literature.^9 20–23^ The CT diagnosis of LAMNs with rupture is relatively straightforward when complex peritoneal diseases and a dilated and ruptured appendiceal mucinous neoplasm are identified in the right lower quadrant.^15 24^ However, the imaging characteristics of LAMNs confined to the appendix have not been well described in the study due to its uncommonness and lack of specific clinical features. Our study showed that all cases were unilocular cystic masses contiguous with base of cecum in right lower quadrant on CT. The mean maximal diameters of masses ranged from approximately 38 to 106 mm with a mean size of 66.3 mm in our series and 12 cases over 60 mm. Although some lesions in our series were very large, all lesions were smaller than 50 mm in width. The ratio of length against width was 1.83 in average in our series in which two cases remained the tubular shape of appendix, probably due to that mucin production and neoplastic expansion were restricted to the appendiceal lumen. LAMNs can appear as cyst-forming masses due to occurrence of cystic or dilatation of the appendix caused by excessive mucin production by mucinous epithelial neoplasm cells which line the inner surface of the cyst. The unenhanced CT values of the intratumoral cystic contents varied from case to case, ranging from 15 to 29 HU with a mean attenuation value of 20.4 HU, which is slightly higher than that of water in this group, probably caused by the presence of high protein and/or mucinous content.^8 25^

As observed in our study, all cystic masses were well demarcated due to the high incidence of fibrous encapsulation which results in a well-circumscribed margin derived from the presence of mural hyalinization and fibrosis.^5 10^ Cyst wall thickness was uneven with a mean value of 5.7 mm, in which most lesions had the minimal wall thickness of 3 mm or less with a focal wall thickening of up to 10 mm in three cases. Owing to the thin and tense cyst wall, it is important for early and accurate preoperative identification because rupture of the mass can lead to free spillage of mucus into the peritoneal cavity at any moment and resultant pseudomyxoma peritonei. In this study, the outer capsule wall was yet regular and smooth, while the inner capsule wall was rough with mural calcification in three cases. Kehagias *et al*^8^ documented on CT scans of a case of LAMN presented as acute appendicitis, a cystic mass with 83 mm in diameter that contained a partly curvilinear mural calcification, which was similar to our cases. Wang *et al*^25^ speculated that mural calcification could be due to a chronic process caused by the mucus in the appendiceal wall. Although this characteristic was very suggestive for mucocele and can be revealed in up to 50% of the cases,^26 27^ the presence of calcification was not indicative of malignancy.^25^ Thus, in a large and thick-walled cystic appendiceal lesion, the diagnosis of a LAMN cannot be excluded despite of the low likelihood of this disease.

As for enhanced CT scan, although no intralesional enhancement was noted in any cases, mildly ring enhancement of the wall was observed in arterial phase. LAMNs were hypovascular tumors and the enhancing rim represented the light vascularity of tumor blood supply in the wall. Moreover, a progressive enhancement in the parenchymal phase reflected an internal fibrillar element and poor venous drainage, which was confirmed in the pathological images. Mini and enhancing mural nodules were observed in five cases. Histological examination indicated that the presence of serrated gland architecture in LAMNs resembled serrated polyp, which was consistent with the present study although it was not prominent.^10^

Differential diagnosis for LAMNs usually includes acute appendicitis, retention cysts and mucinous adenocarcinoma. Due to crossover of symptoms, most of LAMNs are often misdiagnosed preoperatively as acute appendicitis clinically. Because of the different clinical management principles, it is important to differentiate LAMNs confined to the appendix early in the course of the disease preoperatively. Contrast-enhanced CT technique makes it possible to improve the diagnostic accuracy. Acute appendicitis typically appears in CT as enlarged appendixes, appendiceal wall edema, thickening without nodule enhancement and periappendiceal fat stranding.^20 21 28 29^ Yilmaz *et al* 16 concluded that appendicitis occurred at a lower mean age of 36.7 years with a slight male preponderance. Periappendiceal fat stranding was more frequently found in patients with appendicitis, in contrast to low frequency of two cases of LAMNs in our study. Retention cysts were dilatation of the distal appendiceal lumen associated with thinning and atrophy of non-neoplastic mucosa. The wall is usually uniform in thickness and smooth without separation or mural nodules. Lesion diameter generally tends to be <4 cm in retention cyst, while tumors are usually >6 cm in cystadenoma or adenocarcinoma,^30 31^ similar to our cases. In addition, Carmignani *et al* 32 found that 56.1% of 532 patients with mucinous adenocarcinoma showed raised CEA, and 67.1% exhibited raised CA 19-9. These markers are recommended in diagnostic investigations. Therefore, when investigating right lower quadrant pain older females, the present study demonstrated that the thickened cystic wall with ring mural enhancement and a characteristic progressive contrast enhancement were the most discriminating CT criteria for differentiating LAMNs, with an excellent accuracy. Compared with the potential harms associated with the ionizing radiation exposure from CT, given the potential malignant progression, sensitive detection and adequate diagnosis of LAMNs confined to the appendix are more beneficial, will improve the efficacy of the treatment, and allow for better prediction of the prognosis and outcome after therapy.

This study has some limitations which have to be pointed out. The small sample size was the predominant one owing to the rarity of the tumors on which this imaging review was based, followed by a lack of statistical significance. Another limitation was bias on patient selection. The retrospective design of our analysis was based on pathological reports, not on CT findings of an abnormal appendix. The population in the study only included patients with surgically proven LAMNs confined to the appendix at a single center. Additional limitations included some non-specific imaging findings. Nevertheless, LAMNs should be considered when an encapsulated oblong mass is encountered with cystic appearance, including 6 cm or more diameter, slightly higher attenuation than water, 5 mm or more mean wall thickness, small mural nodules or the presence of calcification and the characteristic pattern of progressive enhancement, especially in older females with non-specific symptoms similar to appendicitis.

Preoperative detection of LAMNs confined to the appendix is important because of treatment implications. Although preoperative diagnosis remains challenging, we believe that a combination of clinical manifestations and suggestive findings on CT imaging makes it possible to improve the diagnostic accuracy of appendiceal neoplasms.
